# DCAF-13 is required for *C. elegans* growth, development, and fertility

**DOI:** 10.17912/micropub.biology.000641

**Published:** 2022-09-23

**Authors:** Aidan Durkan, Catherine Byrnes, Emily Cooper, Alyson Hally, Jessica Sullivan-Brown, Jessica Sowa

**Affiliations:** 1 West Chester University of Pennsylvania

## Abstract

DCAF13 (DDB1 and CUL4 associated factor 13) is a potential oncogene but little is understood about the developmental roles of this highly conserved gene. We characterized the RNAi phenotypes of *dcaf-13* , the *C. elegans* homolog of DCAF13, and show that compared to age-matched control worms, body length is decreased in *dcaf-13* (RNAi) *C. elegans* larvae, suggesting a role of *dcaf-13* in larval development. In addition, *dcaf-13* (RNAi) worms display either a failure or delay in reaching the L4 and adult stages. Our data also indicates that *dcaf-13* (RNAi) treatment beginning at L4 stage does not increase embryonic lethality in progeny; however, progeny production was significantly decreased in *dcaf-13* (RNAi) worms, suggesting a general role in fertility and perhaps oocyte development.

**Figure 1. RNAi knockdown of dcaf-13 impairs C. elegans growth, fertility, and development.**
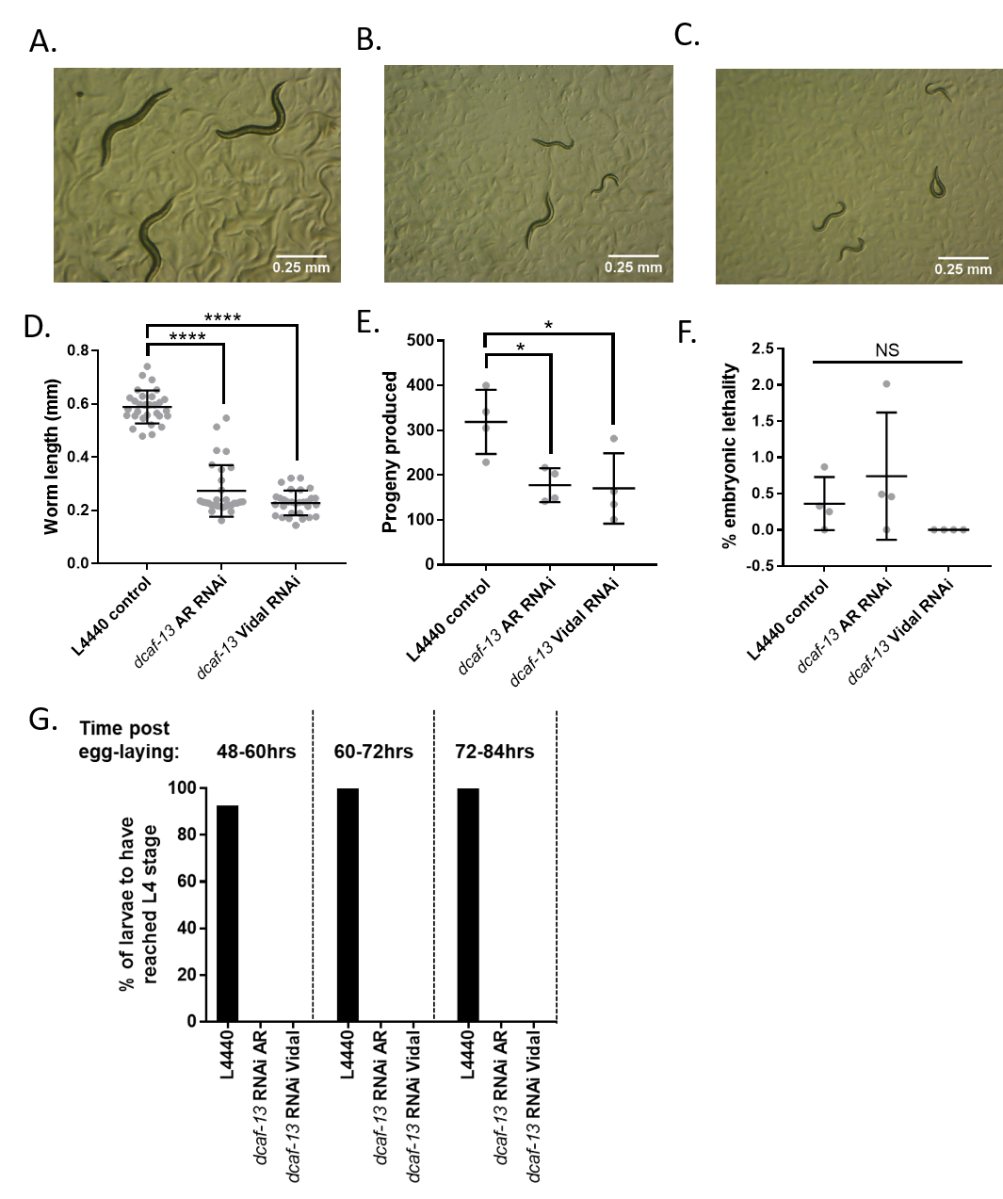
A-C) Representative images of 2-day old A) L4440 control B) Ahringer library *dcaf-13* (RNAi) or C) Vidal library *dcaf-13* (RNAi) treated *C. elegans* . Images were taken at 25X magnification, scale bar=0.25mm. D) Quantification of worm lengths for 2-day old L4440 control or *dcaf-13* RNAi treated *C. elegans* . Graph shows combined data from 3 independent biological replicates, n=10 for each replicate. Treatment with both Ahringer (AR) and Vidal library *dcaf-13* RNAi clones resulted in a significant reduction in worm length compared to L4440 control (One-way ANOVA with Sidak’s multiple comparisons, p<0.001). E) Total progeny produced by 3 adult worms during a 12-hour laying period. L4 stage worms were treated with control or *dcaf-13* RNAi for 24 hours, then allowed to lay eggs for 12 hours before being removed. 24 hours after adult removal, embryos and larvae were counted. Treatment with both AR and Vidal *dcaf-13* RNAi resulted in less progeny production compared to L4440 control treatment (One-way ANOVA with Sidak’s multiple comparisons, n=4, p<0.05). Data shown is from four independent biological replicates. F) Percent embryonic lethality was calculated by dividing the number of unhatched embryos by the total number of unhatched embryos and live larvae produced by RNAi or control treated worms at least 24 hours after egg laying and adult removal. No statistically significant differences in percent embryonic lethality were found (Kruskal-Wallis test, n=4, p=0.08). Data shown is from four independent biological replicates. G) Larvae produced during a 12-hour egg laying window by *dcaf-13* RNAi or control treated *C. elegans* were followed for 72 hours after egg laying and the number of larvae to have reached L4 stage at each time point was recorded. None of the *dcaf-13* (RNAi) larvae had reached L4 stage as of 72 hours post egg laying. Data shown is the average of 2 independent biological replicates. *p<0.05, ****p<0.001

## Description

DDB1 and CUL4 associated factor 13 (DCAF13) is a highly conserved protein with diverse cellular functions. In mammals, DCAF13 is essential for oocyte growth and preimplantation embryonic development (Liu et al., 2019; Zhang et al., 2019, 2018). DCAF13 is a substrate receptor for CUL4-DDB1 E3 ligases, targeting SUV39H1 (a H3K9 methyl transferase) for degradation during mammalian embryogenesis (Lee and Zhou, 2007; Zhang et al., 2018). DCAF13 was also shown to have essential roles in mammalian oogenesis by serving as a nucleolar protein and participating in 18S rRNA processing (Zhang et al., 2019).

In addition to the essential functions of DCAF13 during mammalian oocyte and embryonic development, overexpression of DCAF13 is linked to poorer outcomes for patients with several different types of cancers, suggesting that DCAF13 may serve as an oncogene. Overexpression of DCAF13 was associated with significantly worse prognosis in patients diagnosed with a subgroup of breast cancers (Chin et al., 2007; Wang et al., 2019) hepatocellular carcinoma (Cao et al., 2017; Qiao et al., 2019), and lung carcinoma (Li et al., 2020). Overexpression of DCAF13 has also been associated with cell migration and epithelial-mesenchymal transition in human breast cancer cells (Sun et al., 2022).

Because of the clinical significance of DCAF13 and its’ relatively understudied role in development, we designed experiments to characterize the *dcaf-13* RNAi knockdown phenotypes in *C. elegans* as part of a Cell and Molecular Biology course, providing undergraduates with unique opportunities to perform genuine research and generate novel findings about this gene.

**Results**

**Knockdown of *dcaf-13* decreases *C. elegans* body length**

To determine whether DCAF13 plays a role in *C. elegans* growth and development, we took a feeding RNAi approach to knock down the *C. elegans Dcaf13* homolog, *dcaf-13,* and observed the effects on *C. elegans* growth and development. We focused initially on body length, as this is an easily quantifiable readout for *C. elegans* growth. L4 stage wild type *C. elegans* were treated with L4440 (vector control), Vidal library *dcaf-13* RNAi, or Ahringer library *dcaf-13* RNAi. The progeny of these worms were measured after 48 hours of development on RNAi plates. For each RNAi treatment group, 10 larvae were imaged and body length calculated using ImageJ FIJI. We found that both *dcaf-13* RNAi treatments caused a significant decrease in total body length in three independent biological replicates (Figure 1a-d, One-way ANOVA with Sidak’s multiple comparisons, n=10, p<0.05), indicating that *dcaf-13* is necessary for *C. elegans* growth and/or development.

**Knockdown of *dcaf-13* decreases *C. elegans* fertility but does not cause embryonic lethality**

To determine whether *dcaf-13* is necessary for *C. elegans* fertility and/or embryonic development, we looked at progeny production and embryo viability in *C. elegans* after *dcaf-13* RNAi knockdown. *C. elegans* exposed to *dcaf-13* RNAi from the L4 stage onward produced significantly fewer progeny during a 12-hour timed laying compared to those treated with vector control (Figure 1e, One-way ANOVA with Sidak’s multiple comparisons, n=4, p<0.05), suggesting that *dcaf-13* knockdown impairs fertility. This agrees with a previous report by Zhang et al, which found severe defects in fertility in L4 stage *C. elegans* treated with *dcaf-13* RNAi (Zhang et al., 2019). Interestingly, although progeny production differed between *dcaf-13* RNAi-treated and vector control treated worms, we found no significant difference in embryonic lethality (Figure 1f, Kruskal-Wallis test, n=4, p=0.08).

**Knockdown of *dcaf-13* leads to defects in larval development**

We next wanted to explore whether *dcaf-13* knockdown affects *C. elegans* larval development, or only growth. To evaluate this, we repeated the RNAi treatment used for the length measurements, but this time followed the population of *dcaf-13* RNAi-treated larvae for 72 hours after hatching, checking at 12-hour intervals whether the larvae had reached the L4 stage. L4 stage is easily recognized in *C. elegans* by the distinct morphology of the vulval precursor cells (Corsi et al., 2015). Furthermore, the timing of larval development in *C. elegans* is very uniform; in wild type worms development from L1 to L4 takes an average of 43.5 hours at 20°C (Byerly et al., 1976). We found that both *dcaf-13* RNAi treatments resulted in larval delay or arrest, with none of the *dcaf-13* RNAi treated larvae reaching L4 stage by 72 hours post-laying. This suggests that *dcaf-13* may have unique functions during development and reproduction. In the future, it would be informative to generate null mutants to assess the phenotypes associated with complete removal of *dcaf-13* .

*C. elegans* is a highly advantageous model system for conducting exploratory pathway analysis due to the relative ease of genetic screening and genetic modification in this system; we therefore expect our results to provide the groundwork to apply the power of the *C. elegans* model to better understand the functions and interactions of DCAF13. Conducting these studies in the context of an inquiry-based undergraduate laboratory can provide opportunities for students to experience genuine research while contributing to our understanding of this clinically relevant gene.

## Methods

**RNAi treatment**

RNAi-by-feeding was performed as described in (Kamath et al., 2001). Briefly, RNAi strains L4440 vector control, Ahringer library *dcaf-13* RNAi (Source BioScience), Vidal library *dcaf-13* RNAi (Source BioScience) and Ahringer library *pos-1* RNAi *E. coli* strains were streaked from frozen stocks onto LB plates containing 100µg/ml ampicillin and 10µg/ml tetracycline and grown overnight at 37°C. For each RNAi strain 5ml of LB containing 25µg/mL carbenicillin were inoculated from streaked plates using a sterile pipet tip and grown overnight at 37°C with agitation. 6cm RNAi NGM plates (LabExpress #5003-60) were seeded with 200µL of RNAi overnight culture and left to dry overnight at room temperature. 30 L4 stage N2 *C. elegans* were then transferred to one plate for each RNAi strain. *pos-1* RNAi plates were used to verify RNAi efficacy but not otherwise imaged or analyzed.

**Imaging and length analysis**

*dcaf-13* RNAi and L4440 vector control treatment of L4 worms was performed as described in “RNAi Treatment” above. RNAi treated L4 worms were incubated at 20°C for one day, after which they were transferred to fresh RNAi plates at a density of 3-5 worms per plate. After the transfer, the worms were incubated for one day at 20°C. Then, all worms in the adult stage were removed from the plates and sacrificed, and plates containing embryos were incubated at 20°C for two days prior to imaging. Images were acquired at 25X magnification using a Zeiss Stemi 305 dissecting microscope with a Celestron HD Digital Microscope eyepiece camera using SkyStudioPro software. 10 worms were imaged per treatment group for each replicate of the experiment. Length analysis was conducted using ImageJ FIJI ( https://imagej.net/software/fiji/ ). Mean lengths for each treatment group were compared using a one-way ANOVA with Sidak’s multiple comparisons in Prism 7.05.

**Progeny counts & embryonic lethality**

*dcaf-13* RNAi and L4440 vector control treatment of L4 worms was performed as described in “RNAi Treatment”. After one day of incubation at 20°C on RNAi plates, young adult RNAi-treated worms were transferred to fresh RNAi plates at a density of 3 worms/plate and incubated at 20°C for 12 hours. After this laying period, adults were removed, and plates were placed at 20°C for 24 hours to allow progeny to develop. The total number of live progeny and unhatched eggs on each plate were then counted. Embryonic lethality was calculated as the percent of unhatched eggs out of the total live progeny + unhatched eggs on each plate. Numbers of progeny produced were compared using a One-way ANOVA with Sidak’s multiple comparisons, and rates of embryonic lethality were compared using a Kruskal-Wallis test in Prism 7.05.

**Developmental time analysis**

Progeny from the 12-hour timed laying used for progeny counts and embryonic lethality determination were returned to 20°C after counting. At 48 hours post-laying, plates were checked again and the number of progeny to have reached L4 stage or adulthood were recorded. Plates were re-checked at 12 hour intervals until either all progeny had reached adulthood or 72 hours had elapsed since the end of the laying period. For each check performed, percentage of progeny to have reached L4/adult was calculated by dividing the number of progeny in L4 stage or adulthood by the total number of progeny on the plate. Because eggs may have been laid any time during the 12 hour laying period, times post egg-laying are given as 12 hour ranges.

## Reagents

**Table:** 

**Strain**	**Genotype**	**Available from**
**N2**	***Caenorhabditis elegans***	**CGC**
**Clone CUUkp3301F203Q**	***dcaf-13* Ahringer library RNAi *E. coli***	**Source BioScience (sequence verified)**
**Clone DFClp3320D0810003D**	***dcaf-13* Vidal library RNAi *E. coli***	**Source Bioscience (sequence verified)**
